# Local Application of a New Chalconic Derivative (Chalcone T4) Reduces Inflammation and Oxidative Stress in a Periodontitis Model in Rats

**DOI:** 10.3390/antiox13101192

**Published:** 2024-09-30

**Authors:** Angelo Constantino Camilli, Mariely Araújo de Godoi, Vitória Bonan Costa, Natalie Aparecida Rodrigues Fernandes, Giovani Cirelli, Larissa Kely Faustino da Silva, Letícia Ribeiro Assis, Luis Octavio Regasini, Morgana Rodrigues Guimarães-Stabili

**Affiliations:** 1 Department of Diagnosis and Surgery, School of Dentistry at Araraquara, São Paulo State University (UNESP), Araraquara 14801-903, SP, Brazil; angelo.camilli@unesp.br (A.C.C.); mariely.a.godoi@unesp.br (M.A.d.G.); vitoria.bonan@unesp.br (V.B.C.); natalie.fernandes@unesp.br (N.A.R.F.); giovani.cirelli@unesp.br (G.C.); larissakely93@gmail.com (L.K.F.d.S.); 2 Department of Chemistry and Environmental Sciences, Institute of Biosciences, Humanities and Exact Sciences, São Paulo State University (UNESP), São José do Rio Preto 01049-010, SP, Brazil; leticia.assis@unesp.br (L.R.A.); luis.regasini@unesp.br (L.O.R.)

**Keywords:** periodontitis, chalcone, topical application, antioxidant activity

## Abstract

Chalcones are phenolic compounds with biological properties. This study had the aim to evaluate the effects of topical administration of a new synthetic chalcone, Chalcone T4, in an animal model of periodontitis induced by ligature. Forty rats were distributed in the following experimental groups: negative control (without periodontitis and topical application of distilled water), positive control (periodontitis and topical application of distilled water), chalcone I and II (periodontitis and topical application of 0.6 mg/mL and 1.8 mg/mL, respectively). Chalcone or distilled water was administered into the gingival sulcus of the first molars daily for 10 days, starting with the ligature installation. The following outcomes were evaluated: alveolar bone loss (µCT and methylene blue dye staining), quantification of osteoclasts (histomorphometry), cell infiltrate and collagen content (stereometry), gene expression of mediators (*Nfact11, Tnf-α, Mmp-13, iNos, Sod and Nrf2*) by (RT-qPCR); expression of BCL-2 and Caspase-1 (immunohistochemistry). Chalcone T4 inhibited bone resorption and prevented collagen matrix degradation. Reduction in the expression of inflammatory markers (*Nfact11, Tnf-α, Mmp-13*, and Caspase-1), attenuation of oxidative stress (*iNOS* reduction, and increase in *Sod*), and pro-apoptotic effect of the compound (BCL-2 reduction), were associated its effects on periodontal tissues. Topical application of Chalcone T4 prevented bone resorption and inflammation, demonstrating potential in the adjunctive treatment of periodontitis.

## 1. Introduction

Periodontitis is an inflammatory condition that affects the support and protection tissue of the teeth, and it can cause the reabsorption of the alveolar bone and tooth loss. The pathogenesis of periodontitis involves the disbalance between the biofilm microorganisms and the host immune response. Dental biofilm elicits a host inflammatory and immune response, ultimately leading to the destruction of the periodontium in a susceptible individual [1]. Several studies have demonstrated that the biological activity of the cytokines can directly and indirectly influence the extent and severity of periodontitis [2,3]. According to evidence, an increase in the expression of the inflammatory mediators is found in periodontally diseased sites compared to healthy sites, and the disbalance between the production of pro- and anti-inflammatory mediators is one of the main factors involved in periodontal destruction [2,3]. In addition to the role of inflammatory mediators, the exaggerated production of reactive oxygen species (ROS) associated with a relative deficiency of antioxidants (a condition known as oxidative stress) can cause an increase in oxidative damage in the periodontal tissue, lipid peroxidation in saliva, and gingival fluid [4,5], contributing to the onset and severity of periodontal disease [6]. The production of antioxidant enzymes, necessary to counterbalance the harmful effects of oxidative stress, is in part regulated by the Nrf2 (nuclear factor erythroid 2 related factor 2) signaling pathway [7]. The importance of Nrf2 in the pathogenesis of periodontitis has been demonstrated through in vivo experiments. Nrf2 knockout mice subjected to the experimental model of ligature-induced periodontitis showed the disease more severely in relation to wild-type animals [8].

Besides inflammation and oxidative state, apoptosis has been considered an important factor associated with periodontal destruction [9]. Bacterial microorganisms can cause the apoptosis of gingival fibroblasts of the periodontal ligament, favoring tissue degradation [10,11]. On the other hand, the persistence of inflammatory cells by the induction of anti-apoptotic factors perpetuates the inflammatory process [12].

Considering that the immune–inflammatory response is primarily responsible for the tissue destruction associated with periodontitis, there is great interest in the use of immunomodulatory agents, which have the potential to prevent/reduce bone resorption and connective tissue destruction. Chalcones are a specific class of natural compounds and synthetic prototypes with different biological effects (anti-inflammatory, antioxidant, anti-cancer, and anti-resorptive action) and high immunomodulatory potential [13,14,15]. Due to their ability to act on a series of targets of the inflammatory process, involving transcription factors, adhesion molecules, intracellular signaling pathways, and inflammatory mediators [13,16], these phenolic compounds have shown to be a promising therapeutic alternative for inflammatory diseases as inflammatory bowel diseases, rheumatoid arthritis, sepsis [17,18,19,20], and osteolytic pathologies (bone cancer and osteoporosis) [21,22,23]. Preclinical studies have demonstrated the effects of systemic administration of different chalconic derivatives on the reduction of clinical signs and symptoms of inflammatory diseases such as rheumatoid arthritis [24,25] and inflammatory bowel diseases [26,27] via the negative modulation of inflammatory mediators (inducible nitric oxide synthase enzyme (iNOS), cyclooxygenase-2 (COX-2), tumor necrosis factor alpha (TNF-α), interleukin 6 (IL-6)) [28], and attenuation of oxidative stress by the increase in antioxidant enzymes (superoxide dismutase (SOD) and glutathione (GSH)), and reduction of reactive oxygen species (ROS) [15]. 

Regarding anti-resorptive activity, the chalcone has shown the capacity to inhibit the differentiation and osteoclast activity in vitro [19,29] and the in vivo bone resorption [23,29] and suppress the expression and activation of transcription factors (factor of activated T cells cytoplasmic-1 (NFATc1)), nuclear factor kappa B (NF-kB), and inflammatory mediators (matrix metalloproteinase-9 (MMP-9), cathepsin K, receptor activator of nuclear factor kappa B ligand (RANKL), TNF-α), responsible for regulating bone resorption [19,29]. 

In view of the various results demonstrating the anti-inflammatory and anti-resorptive effect of chalcones, our research group evaluated the effects of topical administration of a new chalcone (Chalcone T4) in a model of experimentally induced periodontitis in rats [29]. Corroborating the aforementioned results, our previous data demonstrated that oral administration of Chalcone T4 significantly reduced bone resorption and the inflammatory process in periodontally diseased tissues without causing any secondary effects [29]. Although numerous studies have evaluated the effects of different chalcones systemically administrated, there is a lack of data on the effects of local application of these compounds in modulating periodontitis. Considering the site-specific characteristic and inflammatory nature of periodontal disease and the antiresorptive and anti-inflammatory properties of Chalcone T4, this research evaluated for the first time the effects of topical application of chalcone on the progression of experimental periodontitis. 

## 2. Materials and Methods

### 2.1. Chalcone 

Chalcone T4 was synthetized at the Laboratory of Medicinal Chemistry, Department of Chemistry and Environmental Sciences, São Paulo State University (UNESP), São José do Rio Preto, Brazil. The synthesis of the substance was carried out by means of the Claisen–Schmidt condensation reaction, with satisfactory yields (50–70%). Recrystallization and chromatographic techniques were used to purify the substance, including normal-phase column chromatography (silica gel), reverse-phase column chromatography (octadecylsilane), and gel permeation chromatography (LH-20). The structure of the compound was identified by mass spectrometry and hydrogen and carbon-13 nuclear magnetic resonance techniques (1H NMR and 13C NMR). The purity of the substance was determined by high-performance liquid chromatography with photodiode array detection (HPLC-DAD) analysis, showing values equal to or greater than 95.0% [29]. Chalcone T4 was stocked at 4 °C, protected from light, and diluted in 1% carbopol to acquire a gel consistency.

### 2.2. Experimental Design and Periodontitis Induction

The experimental procedure was submitted and authorized by the Ethical Committee for Animal Use (CEUA) of the School of Dentistry at Araraquara, UNESP (permit number: 23/2020) and realized in agreement with the guidelines from the National Council for Animal Experiment Control (CONCEA). A total of 40 rats (Rattus norvegicus albinus, Holtzman), males weighing 150–200 g, approximately 4 weeks old, were used in this study. The animals were randomly divided into 4 experimental groups (10 animals per group), according to the presence or absence of periodontitis and the compound administered: positive control group (ligatures and application of distilled water (Ø+)), negative control (no ligatures and application of distilled water (Ø−)), chalcone I and II (ligature and topical application of 0.6 mg/mL (Chal I) and 1.8 mg/mL (Chal II) of Chalcone T4), respectively. A vehicle group was not added to this methodology since preliminary results from our group demonstrated the absence of biological effects of the vehicle on bone loss and inflammation in a rat study using the periodontitis model [30]. The sample size calculation was based on a study with a similar methodology, whose primary denouement is change in bone volume in model periodontitis in rats [31]. All animals in the ligature-induced group were anesthetized with ketamine (80 mg/kg) and xylazine (7 mg/kg) for periodontitis induction. The ligatures were placed using a silk thread (Ethicon, Johnson & Johnson, Somerville, NJ, USA) tied around the cervix of the first lower molars bilaterally. In all animals, the ligature was inspected every other day and repositioned if necessary to maintain the ligature during the entire experimental period.

### 2.3. Administration of Chalcone T4 and Sample Collection

The Chalcone T4 was diluted in 1% carbopol and used in two concentrations (0.6 mg/mL and 1.8 mg/mL). The selected concentrations were based on a previous study [30] that demonstrated a reduction in bone resorption in rats that received topical application of 0.6 mg/mL of a chalconic compound associated with essential oil in a periodontitis model. In the present study, in addition to the concentration of 0.6 mg/mL, the effect of a concentration 3 times higher (1.8 mg/mL) was also determined in order to evaluate a possible dose-response effect. A 30 µL amount of each dose of Chalcone T4, or distilled water, was applied topically to the gingival sulcus around the first molars using a sterile 1 mL disposable syringe and a 13 × 0.38 mm needle (INJEX, Ourinhos, SP, Brazil). Applications were performed daily, once a day, for 10 days, starting on the same day as the ligatures were installed. For the applications, the animals were submitted to an inhalation method using isoflurane in an adapted vaporizer (Fluovac Anesthesia System for Rats with Induction Box (Harvard Apparatus, Holliston, MA, USA)). Ten days after the insertion of ligatures, the rats were euthanized by a lethal anesthetic dose, and the jaws were removed and split into two hemimandibles. The gingival tissue around the ligatures from the right hemimandibles was collected and processed for molecular analysis (RT-qPCR), and the hemimandibles were kept in 70% ethanol for methylene blue dye staining microcomputed tomography (μCT). The hemimandibles on the left side were decalcified in 0.5% ethylenediaminetetraacetic acid (EDTA), pH 7.2 for 5–6 months, embedded in paraffin and used in histological and immunohistochemical analyses.

### 2.4. Methylene Blue Dye Staining

The right hemimandible was removed from the 70% ethanol and kept immersed at 29% hydrogen peroxide for 3 h to permit the manual removal of all the soft tissue. The samples were stained with a solution containing 3.5 mg/mL of methylene blue dye for 2 min. The stained samples were positioned in a standardized manner in a stereomicroscope (Leica HZ6—1.25× magnification, Wetzlar, Germany), and images of the vestibular aspect were captured. The first lower molar was considered to measure the area of bone loss between the cement enamel junction and the alveolar bone. The area stained with methylene blue corresponds to the exposed root surface (dental enamel is not stained with methylene blue) and directly corresponds to the extent of bone resorption, which was analyzed histomorphometrically using the ImageJ program (NIH Image (v.1.51s, National Institutes of Health, Bethesda, MD, USA—http://imagej.nih.gov/ij, accessed on 9 September 2024)). The analysis was performed by a single trained examiner, who was not aware of the experimental group allocation of the specimens, as described by Fernandes et al. [29].

### 2.5. Microcomputed Tomography Scanning (μCT)

The hemimandibles were scanned on a μCT imaging system (Skyscan 1076; Bruker, Kontich, Belgium) using 9 μm (0.01 mm) isotropic spatial resolution, 0.5 mm aluminum filter, 80 kV voltage, 310 μA current, exposure of 195 ms, frame average of 3 and rotation step of 0.4° in 180°. The parameters for reconstruction (NRecon 1.6.1.5; Bruker) and the limits of the bone region of interest (ROI) were established as described by Fernandes et al. [29]. The ROI was delimited to every 10 slices, in a total of 90 slices. The percentage of bone volume fraction was quantified in the CT Analyzer 1.15.4.0 software ((BVF)%). 

Linear bone loss was measured by calculating the distance between the cementum–enamel junction (CEJ) and the alveolar bone crest (ABC) on the distal surfaces of the first molars and mesial surfaces of the second molars. The analyses were carried out by a researcher previously calibrated and unaware of the experimental groups. Results are representative of 5 animals per group.

### 2.6. Histometric and Stereometric Analysis

Histological sections (4 µM of thickness), with intervals of 80 µM were obtained in the sagittal plane. For the histometric analysis, the slices were stained with Masson’s trichrome. Giant and multinucleated cells (with 3 or more nuclei) in proximity to the bone surface, located in resorption gaps in the furcation region of the first molar, were considered osteoclasts.

For the stereometric analysis, the number of cells (inflammatory and fibroblasts, without distinction) and collagen fibers were determined by superimposing a grid on the histological image of a region of interest. The region of interest was determined for each slice, delimited by the following anatomical structures: coronally by the most apical portion of the epithelial tissue, apically by the top of the bony crest, and laterally by the most prominent region of the distal root of the first molar and by the mesial root of the second molar. The images were obtained by an optical microscope coupled to a color digital camera using standardized settings for image acquisition (Leica Application Suite 3.8, Wetzlar, Germany) (200×). For each slice, two grids were needed, each containing 90 points of intersection, and the proportion of tissue components in the area of interest was obtained in relation to the total number of points counted. At least 3 sections per animal were analyzed from at least 4 animals per group. All analyses were performed by a trained examiner blinded to the experimental groups.

### 2.7. Immunohistochemistry

For analysis of BCL-2 and Capase-1 expression and immunolocalization, the histological sections were mounted on silanized slides and submitted to immunohistochemistry reaction using specific primary antibodies (Santa Cruz Biotechnology, Dallas, TX, USA, # sc-783 and Abcam, Cambridge, UK, # ab-1872, respectively). Negative control sections were incubated with PBS (with the absence of the primary antibody) to evaluate background staining. Dako Envision^TM^ FLEX visualization kit (Glostrup, Denmark), high pH (Link)–(#K800021-2), was used to visualize the target protein. The sections were counterstained with Carrazi’s hematoxylin and mounted with Permount. Images at a magnification of 40× were obtained using a microscope (LEICA microsystem GmbH, Wetzlar, Germany) coupled with a digital color camera. The region of interest analyzed was the same as described for stereometric analysis. The results were analyzed by an examiner without knowledge of the experimental groups, using the ImageJ software (v.1.51s, National Institutes of Health, USA—http://imagej.nih.gov/ij, accessed on 9 September 2024) and the IHC Profile plugin, as described by Nguyen DH 2013 [32]. This analysis is based on the determination of intensity and area of chromogen staining. Slides of at least 4 animals per group were evaluated. The results were used to determine an average value per animal, and then these values were compared between groups. 

### 2.8. RT-qPCR

Total RNA of the gingival tissue around the first molar was extracted with Trizol reagent (Invitrogen Corp., Carlsbad, CA, USA) following the manufacturer’s orientations. The purity and amount of RNA were measured in a spectrophotometer by reading the absorbance at 260 nm and the relationship between the absorbances at 260 and 280 nm, respectively. A 700 ng amount of RNA was used for cDNA synthesis using random hexamers as primers and following the reagent supplier’s instructions (high-capacity cDNA synthesis kit, Applied Biosystems, Foster City, CA, USA). The expression of selected inflammatory genes was determined by real-time RT-qPCR using Taqman probes and reagents (TaqMan Gene Expression Assays, TaqMan Universal master mix, Applied Biosystems) in a StepOne Real-Time PCR system (Applied Biosystems). Reactions were performed in 96-well plates with a final volume of 10 µL that included Taqman Universal PCR Master Mix (Applied Biosystems), Taqman Gene Expression Assays (Applied Biosystems) for each gene: (Nfatc1, Tnf-α, Mmp-13, iNOS, Sod, Nrf2 GAPDH) (Table 1). Expression of the genes was determined using the ∆CT comparative method available in the thermocycler software (StepOne v 2.3). For each sample, gene expression analyses were performed in duplicate. Samples from at least 4 animals per experimental group were used.

### 2.9. Statistical Analysis

Data from individual experiments were evaluated using GraphPad Prism 8.0 (GraphPad Software Inc., La Jolla, CA, USA). The distribution of the data generated in this study was assessed using the Shapiro–Wilk normality test and ANOVA with Tukey’s post hoc test for paired comparisons. The significance level was set at 95% (*p* < 0.05) in all analyses.

## 3. Results

Topical application of Chalcone T4 did not alter the integrity of the gingival tissues or cause any side effects in the animals, compared to the control groups.

### 3.1. Chalcone T4 Reduced Bone Resorption

**Figure 1:**
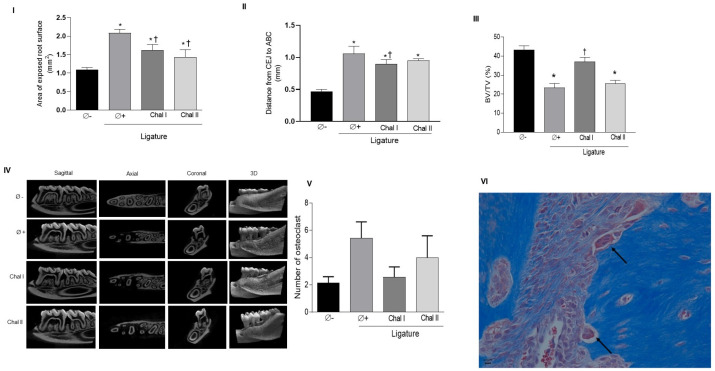
Chalcone T4 (0.6 mg/mL and 1.8 mg/mL) reduced the exposed root surface area of the mandibular first molar, indicating a reduction of bone resorption (**I**). Chalcone T4 (0.6 mg/mL) decreased the distance between CEJ and ABC (**II**) and the volume of bone tissue in the ROI in the ligature-induced periodontitis model (**III**). Results are representative of 8 animals per experimental group. Representative images of each experimental group acquired in the sagittal, axial, coronal, and 3D planes (**IV**). The bar graph indicates the number of osteoclasts in a defined region (furcation of the first molar and interproximal region between the first and second molars) (**V**). Image of the ABC region between the first and second molars. Arrows indicate osteoclasts identified by morphology and location in histological sections stained with Masson’s trichrome (at a 40× magnification 40×) (**VI**). The bars illustrate the mean values, and the vertical lines the standard error of the mean (SEM). (*) (*p* < 0.05) compared to Ø− group. (†) (*p* < 0.05) compared to the Ø+ group.

The results of methylene blue staining demonstrated that the installation of ligatures induced relevant bone loss and that both groups treated with Chalcone T4 showed a significant reduction in bone loss compared to the positive control group (Ø+) (Figure 1I). Using the microcomputed tomography analysis, we also observed a greater bone loss demonstrated by the increase in the distance between the CEJ and the ABC (Figure 1II), and a smaller proportion of bone tissue within the region of interest (furcation of the first molars) in the animals subjected to ligature (50% lower in relation to animals without ligature) (Figure 1III,IV), indicating that the experimental model was effective in inducing alveolar bone resorption during the 10-day experimental period. Interestingly, in this analysis, only the topical application of the lowest dose of chalcone significantly prevented experimentally induced bone loss, as evidenced by the disparity in mean bone volume fraction between the Chal I group and the positive control group (Ø−) (37% vs. 23%, respectively), and distance between the CEJ and the ABC in the different groups (Figure 1II,III). The quantification of osteoclasts through histometric analysis (Figure 1V,VI) indicated an increase in the number of osteoclasts in the positive control group compared to the periodontally healthy group (*p* < 0.05). The application of chalcone reduced the number of osteoclasts in relation to the positive control group (53% reduction); however, this difference did not reach statistical significance (*p* > 0.05).

### 3.2. Topical Application of Chalcone T4 Preserves Collagen Content in the Gingival Tissues

**Figure 2:**
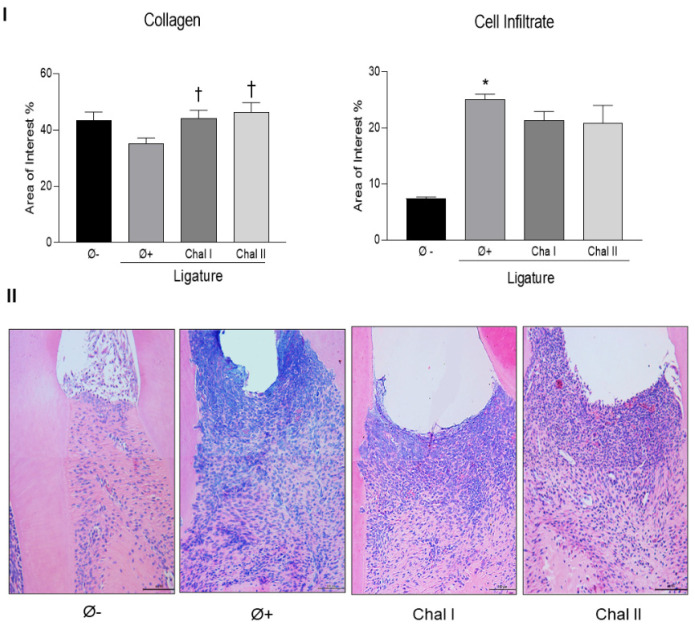
Chalcone T4 inhibits collagen fiber degradation (**I**). A grid was overlaid on the digitized images of the interest area to conduct a quantification analysis of tissue components (cellular infiltrate and collagen fibers) relative to the total number of points counted. Chalcone T4 did not alter the cell infiltrate but prevented collagen content destruction (**I**). Histological appearance of the studied group’s experimental groups (200× magnification) (**II**). The bars represent the mean percentage value, and the vertical lines denote the standard error of the mean (SEM). (*) *p* < 0.05 compared with the Ø− group. (†) *p* < 0.05 compared to the Ø+ group.

Stereometric analysis data demonstrated that periodontitis induction increased the cellular infiltrate and decreased the proportion of collagen fibers, as indicated by the changes between the positive and negative control groups, and that the application of both doses of Chalcone T4 markedly prevented the degradation of collagen fibers (% collagen fiber density in the region of interest: positive control vs. Chal I vs. Chal II: 32% vs. 44% vs. 46%) (Figure 2I). The data in Figure 2I also indicate that Chalcone T4 slightly reduced the cellular infiltrate in relation to the positive control group, however, without a statistically significant difference (*p* > 0.05). The representative images of the stereometric analysis are illustrated in Figure 2II.

### 3.3. Chalcone T4 Reduces the Expression of Inflammatory Markers, Modulates Mediators of Oxidative Stress, and Inhibits Anti-Apoptotic Factor

**Figure 3:**
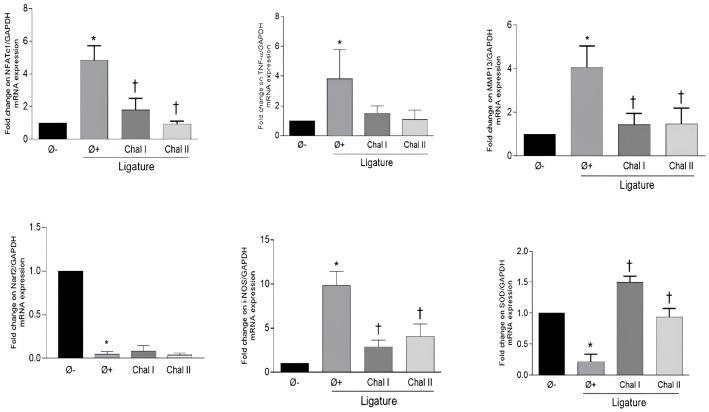
Administration of Chalcone T4 inhibited the expression of inflammatory genes *Nfact1*, *Mmp-13*, and *iNOS* and increased the expression of an antioxidant gene *Sod* in the gingival tissues. Data from RNA extracted from gingival tissues from at least four hemi-mandibles of individual animals from each experimental group. The bars illustrated the mean values, and the vertical lines the standard error of the mean (SEM). (*) *p* < 0.05 compared with the Ø− group. (†) *p* < 0.05 compared to the Ø+ group.

**Figure 4:**
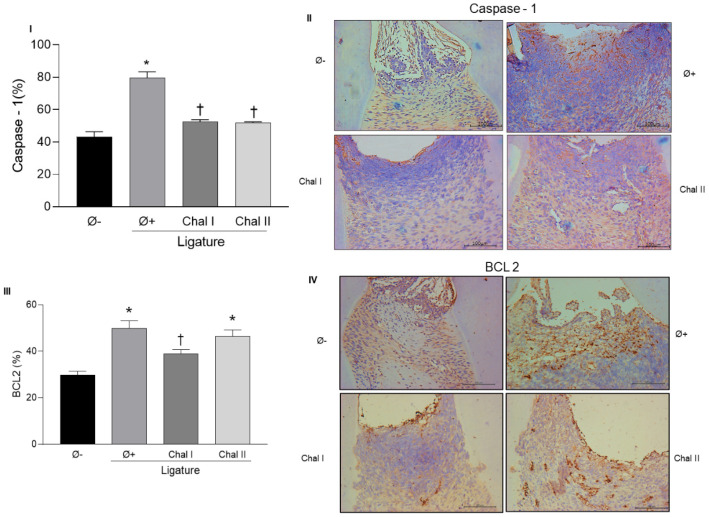
Chalcone T4 (0.6 mg/mL and 1.8 mg/mL) reduced Caspase-1 expression in the region of interest (**I**,**II**). Chalcone T4 (0.6 mg/mL) reduced the expression of BCL-2 (**III**). Images (**II**,**IV**) show the immunohistochemical staining of the studied groups (200× magnification). The bars indicate the mean percentage value, and the vertical lines indicate the standard error of the mean (SEM). * *p* < 0.05 compared to the Ø− group. † *p* < 0.05 compared to the Ø+ group. Scale bar (100 µM).

To evaluate the effect of the topical application of Chalcone T4 on the expression of inflammatory mediators and to correlate with the results found in the µCT and stereometric analysis, we investigated the gene expression of *Nfatc1, Tnf-α, Mmp-13, iNos, Nrf2,* and *Sod* in gingival tissues of animals from different groups, by RT-PCR (Figure 3), and the Caspase-1 protein levels (Figure 4I,II) by immunohistochemistry. Periodontitis induction increased the expression of all markers, with the exception of the antioxidant enzyme *Sod* and the transcription factor *Nrf2*, which were reduced in the periodontally diseased group (group positive control). The application of Chalcone T4, at both doses, significantly decreased the expression of inflammatory mediators *Nfatc1*, *MMP-13*, and *iNOS* and increased the expression of *Sod* (*p* < 0.05). 

To investigate the hypothesis that the anti-inflammatory effect of Chalcone T4 could also result from a pro-apoptotic action of the compound on inflammatory cells, we evaluated the expression of an anti-apoptotic marker, BCL-2, in the gingival tissues. The results of the immunohistochemical analysis showed an increase in the expression of BCL-2 in the animals with periodontitis and a reduction in the animals treated with the lowest dose of the compound (*p* < 0.05) (Figure 4III,IV).

## 4. Discussion

In the current research, we investigated for the first time the biological effect of the topical application of a synthetic chalcone on the evolution of periodontitis in an experimental model in rats. The results indicated that Chalcone T4 reduced bone resorption and prevented collagen matrix degradation. Reduction in the expression of inflammatory markers, modulation of oxidative stress, and the pro-apoptotic effect of the compound were associated with its effects on periodontal tissues.

Plenty of preclinical research has demonstrated the anti-inflammatory and antiresorptive effect of chalcones in inflammatory bone diseases, such as rheumatoid arthritis [33,34,35], osteoporosis [36,37], and bone tumors [38,39]. The compound’s ability to negatively modulate the expression of inflammatory mediators (RANKL, IL-1β, MMP-2, MMP-8, TNF-α, NO, PGE2) and transcription factors (NFATc1, NF-KB) related to inflammatory bone resorption, has been correlated with the effects observed [38].

Corroborating the scientific data showing the anti-osteolytic potential of chalcones, our research group previously demonstrated that systemic administration of Chalcone T4 prevented tissue inflammation and bone resorption induced by periodontitis in rats [29]. Reduction in TNF-α levels and inhibition of NF-kB activation were also observed [29].

In the present study, we demonstrated that topical application of Chalcone T4 was also able to inhibit ligature-induced alveolar bone loss using two complementary methodologies: methylene blue dye staining and microcomputed tomography. Interestingly, the results of methylene blue staining demonstrated that both doses of chalcone reduced the area of the exposed root surface, indicating the antiresorptive effect of the compound. The microcomputed tomography data, however, demonstrated a greater volume of bone tissue in the furcation region of the first molars of animals treated only with the lowest dose of Chalcone T4. The lack of correspondence between the results obtained with the different techniques is probably related to the methodological differences between them: methylene blue staining is a bidimensional technique (allows visualization of two planes (length and width)) and is based on staining the root surface with methylene blue, to allow delimitation of the area of the exposed root surface. Microcomputed tomography, on the other hand, is a three-dimensional technique (that is, it allows visualization in three planes: length, width, and thickness (volume)). The results obtained from µCT, presented in this study, are in line with those found with the systemic administration of Chalcone T4 [29], in which only the lowest dose of the compound was effective in modulating inflammatory bone resorption. Several factors may be involved in this result, such as variations in the solubility coefficient or in the absorption levels of each concentration of the compound, which may have prevented the achievement of sufficient levels for the compound to modulate other relevant targets of the bone resorption process.

The results obtained also demonstrated that Chalcone T4 reduced the expression of Nfatc1, Mmp-13, TNF-α, iNOS, and Caspase-1 and increased the expression of Sod in the gingival tissues. These mediators were evaluated because they are related to the pathogenesis of periodontal disease and because they actively participate in the process of osteoclastogenesis and tissue degradation. NFATc1 is a key transcription factor for osteoclast differentiation and expression of genes that are involved with the formation and activity of these cells [40]. TNF-α, one of the main players in inflammatory bone resorption, can increase the number of osteoclast precursor cells and the expression of RANKL in immune and stromal cells, in addition to decreasing osteoblast differentiation [41]. In inflammatory scenarios, TNF-α can also stimulate the autocrine and paracrine production of MMP-13, which, in addition to its destructive proteolytic activity, can act directly on the formation and activity of osteoclasts, favoring the development of osteolytic lesions [42]. In addition to the expression of these mediators, oxidative stress, represented by the increase in the production of reactive oxygen species and oxidant enzymes, has demonstrated destructive effects on periodontal tissues, stimulating the differentiation and activity of osteoclasts and increasing the severity of periodontitis [43,44]. Considering the role of these biological mediators in the progression of inflammation and bone resorption, it is possible that modulation of these genes by the compound may be involved in the protective role of Chalcone T4 on the progression of periodontitis.

In addition to the suppressive effects of the compound on inflammatory gene expression, the results demonstrated that Chalcone T4 had a preventive effect on the degradation of the extracellular matrix. The experimental model of ligature-induced periodontitis used in this study stimulates the establishment of a dysbiotic oral microbiota, which, in addition to causing direct tissue damage, elicits an immune–inflammatory response characterized by the overexpression of inflammatory mediators and proteolytic enzymes capable of causing the degradation of the extracellular matrix and collagen fibers in periodontal connective tissue. In this context, matrix metalloproteinases, such as MMP-13, stand out for their potent collagenolytic activity and correlation with the activity and severity of periodontal disease [45,46]. Previous results have shown that MMP-13 silencing in the periodontally diseased gingival tissues of rats was related to a lower extracellular matrix degradation and a higher proportion of collagen content [47]. In view of the scientific evidence, the results of the present research indicate that the inhibition of Mmp-13 by the compound is associated with the preventive effect of Chalcone T4 on the destruction of collagen content.

In addition to inflammation regulated by cytokines, intracellular signaling pathways, and oxidative stress, apoptosis has also been considered an important factor for periodontal destruction [9,11]. During tissue inflammation, temporal activation of pro- and anti-apoptotic factors plays a relevant role in the pathogenesis of the disease. Bacterial pathogens induce apoptosis of immune cells, particularly neutrophils, in the periodontal ligament [11]. In response to the increase in cellular apoptosis in inflamed tissues, there is an increase in the expression of anti-apoptotic markers, such as BCL-2, which inhibit the apoptosis of inflammatory cells, favoring the chronification of the inflammatory process [48]. In fact, the results showed an increase in the expression of BCL-2 in the gingival tissue of periodontally diseased animals (positive control group) in the present study. Topical administration of a lower dose of Chalcone T4 reduced the expression of BCL-2, indicating that the compound can modulate apoptotic mechanisms implicated in the inflammatory process associated with gingival tissue destruction.

Caspase-1 is an inflammatory enzyme activated through the assembly of the inflammasome in response to both pathogen-derived and endogenous mediators. Its major effect is to cleave the preforms of inflammatory cytokines IL-1β [49]. IL-1β is an important pro-inflammatory cytokine in the pathogenesis of periodontal disease since it is responsible for activating endothelial cells and facilitating the adhesion of eosinophils, thereby increasing the inflammatory response. IL-1β is also associated with the reabsorption of the alveolar bone by stimulating osteoclast differentiation and activity [50]. Our results showed that the topical application of Chalcone T4 reduced the expression of Caspase-1 in the gingival tissue of the animals. The effect of Chalcone T4 on Caspase-1 downregulation may be related to the preventive effects of the compound on bone resorption and matrix degradation.

In this study, we advanced the previous findings by demonstrating that topically applied Chalcone T4 is also effective in preventing tissue inflammation and bone resorption, which are hallmarks of periodontitis. These results are interesting since they open up possibilities for topical use of the compound, which is particularly relevant in site-specific diseases such as periodontitis. Another important aspect to highlight is that the benefits found by the compound in controlling disease progression were obtained from the use of the lowest dose, which may be an advantage because it indicates that future applications using this compound and dose are more economical and less prone to side effects or off-target effects.

Collectively, our data indicate that topical administration of Chalcone T4 prevented bone resorption and reduced extracellular matrix degradation in an experimental periodontitis model, indicating its potential to be used in the adjunctive treatment of periodontitis. It is important to highlight that although the ligature model used in this study is not a perfect model and does not involve the full complexity of periodontitis in humans, it is capable of inducing the major hallmarks of periodontitis, such as the presence of inflammatory infiltrate in the tissue gingival, connective tissue degradation, and alveolar bone resorption.

Considering the results obtained in this study and its limitations, additional studies that evaluate the biological effects of lower concentrations of the chalcone, and different vehicles for dilution of compounds, such as nanoformulations or lipid vehicles, with the potential to increase the bioavailability and tissue absorption of the compound, should be conducted, as well as studies that evaluate the use of Chalcone T4 in other models of inflammatory diseases.

## 5. Conclusions

The results of this study indicate that topical application of Chalcone T4 prevented alveolar bone loss and inflammation in periodontal tissues, indicating that the compound may be useful in the adjunctive therapy of periodontitis and/or other inflammatory osteolytic diseases.

## Figures and Tables

**Table 1:** References of gene expression assays (primers and pre-designed probes) of the TaqMan system used.

Gene	Accession Number
*GAPDH*	NM_001289726
*Nfatc1*	NM_001164109
*Tnf-α*	NM_013693
*Mmp-13*	NM_008607
*iNOS*	XM_006246949
*Sod*	NM_011434
*Nrf2*	AH006764

## Data Availability

All of the data is contained within the article.
